# Virulence traits associated with *Burkholderia cenocepacia* ST856 epidemic strain isolated from cystic fibrosis patients

**DOI:** 10.1186/s13756-017-0215-y

**Published:** 2017-06-06

**Authors:** Milka Malešević, Zorica Vasiljević, Aleksandar Sovtić, Brankica Filipić, Katarina Novović, Milan Kojić, Branko Jovčić

**Affiliations:** 1 Institute of Molecular Genetics and Genetic Engineering, University of Belgrade, Vojvode Stepe 444a, P.O. Box 23, Belgrade, 11010 Serbia; 2 Department of Clinical Microbiology, Mother and Child Health Care Institute of Serbia “Dr. Vukan Cupic”, Radoja Dakica 8, Belgrade, 11070 Serbia; 3 Department of Pulmonology, Mother and Child Health Care Institute of Serbia “Dr. Vukan Cupic”, Radoja Dakica 8, Belgrade, 11070 Serbia; 4 School of Medicine, University of Belgrade, Dr Subotica 8, Belgrade, 11000 Serbia; 5 Faculty of Pharmacy, University of Belgrade, Vojvode Stepe 450, Belgrade, 11221 Serbia; 6 Chair for Biochemistry and Molecular Biology, Faculty of Biology, University of Belgrade, 16, Studentski trg, Belgrade, 11000 Serbia

**Keywords:** *Burkholderia cenocepacia* complex, Cystic fibrosis, Epidemic strain, Virulence

## Abstract

**Background:**

*Burkholderia cenocepacia* is considered one of the most problematic cystic fibrosis (CF) pathogens. Colonization prevalence in the Serbian CF population is high and virtually exclusively limited to a single highly transmissible clone of *B. cenocepacia* ST856 which is positive for both the *B. cepacia* epidemic strain marker (BCESM) and cable pilin, and is closely related to the epidemic strain CZ1 (ST32).

**Methods:**

Biofilm formation for 182 isolates, and adhesion to components of the host extracellular matrix, proteolytic activity, mucoidy and motility of selected ST856 representatives, as well as *B. cenocepacia* ST858 and ST859, and *B. stabilis* ST857, novel STs isolated from Serbian CF patients, were investigated in this study. The presence of the *cepI*, *cepR*, *fliG*, *llpE*, *wbiI*, and *bcscV* genes was analyzed.

**Results:**

Biofilm-formation ability of analyzed strains was poor under standard laboratory conditions, but changed in stress conditions (cold stress) and conditions that mimic CF milieu (increased CO_2_). All strains expressed ability to bind to collagen and fibronectin albeit with different intensity. Representatives of ST856 exhibited gelatinase activity. ST858, ST859 and 9/11 of ST856 genotypes were positive for swimming and twitching motility whereas ST857 was non-motile. Mucoidy was demonstrated in all ST856 genotypes, ST857 was semi-mucoid, and ST858 and ST859 were non-mucoid. Molecular analysis for major virulence factors revealed that ST856 and ST857 carried the six analyzed genes, while ST858 and ST859 were negative for the *llpE* gene.

**Conclusion:**

Variations in virulence phenotypes in different genotypes of epidemic *B. cenocepacia* ST856 clone, in vitro, could be a consequence of diversification driven by pathoadaptation. Diversity of epidemic clone genotypes virulence, could be challenging for accurate diagnosis and treatment, as well as for infection control.

## Background

Bacteria of the *Burkholderia cepacia* complex (Bcc) have been considered among the most challenging pathogens involved in cystic fibrosis (CF) lung disease [1]. Disparate outcomes of Bcc infections in CF vary from asymptomatic carriage to progressive respiratory deterioration or even rapidly fatal and uncontrollable “cepacia” syndrome [2]. *B. cenocepacia* and *B. multivorans* are currently the Bcc species most frequently isolated from clinical samples [3, 4]. Notably, *B. cenocepacia* comprises the most virulent and transmissible epidemic clones. Strains belonging to this species had often been reported in association with poor clinical outcome and high mortality among CF patients [1]. During infection of CF patients, Bcc bacteria experience stressful conditions, host immune defense, antimicrobial therapy, nutrient availability and oxygen limitation. Although the process of adaptation to the airways of CF patients is still poorly understood it could be attributed to the undeniable virulence potential of *B. cenocepacia* and assumption that these bacteria may encode more potent or more numerous virulence factors comparing to other Bcc species [5]. Among those are LuxIR homolog CepIR (quorum sensing) that was shown to be widespread in Bcc bacteria and required for full virulence in many Bcc infection models, T3SS, motility (flagellum) and efflux pumps that enhance the process of colonization, adaptation and infection [5]. Bacterial virulence factors are delivered either in the extracellular environment or directly into host cells. Most Gram-negative CF pathogens possess one or more specialized secretion systems to accomplish this task, among which is T3SS. A T3SS mutant of *B. cenocepacia* was attenuated in virulence in a murine model of infection, which indicates a role for the T3SS in evasion of the host immune system [6]. Also, there are some unique aspects of efflux systems in *Burkholderia* species that are without parallel. The first gene in the *bpeEF-oprC* operon, *llpE*, was shown to be the most prevalent in *B*. *cenocepacia*, and is co-transcribed with the genes encoding the BpeEF-OprC efflux pump components that was shown to be of importance for resistance to antibiotics [7, 8]. Additionally, the O-antigen portion (coded by the *wbiI* gene) of the LPS molecule is important for resistance of Bcc to serum-mediated killing [9].

However, many of the virulence factors characterized in *B. cenocepacia* appear not to be unique to this species and have homologs in other bacterial pathogens. The detection of two putative epidemic and virulence markers, cable pili [10] and *B. cepacia* epidemic strain marker (BCESM), had been of particular interest and used to be applied as an infection control measure in limiting the spread of infamous *B. cenocepacia* ST28 [3, 5, 11]. However, later evidence suggested that neither ‘marker’ is an accurate indicator of transmissibility or virulence [12]. The *cblA* gene is found almost exclusively in strains of the ST28 while BCESM occurs in many different strains from *B. cenocepacia* [11].

Our previous study evaluated the prevalence and epidemiological characteristics of Bcc strains in the National CF Centre in Belgrade, Serbia [13]. In this study, three novel STs of *B. cenocepacia* were described (ST856, ST858, and ST859) as well as a novel *B. stabilis* strain, ST857 [13]. *B. cenocepacia* ST856 was distinctly dominant and was found in 96% patients infected with Bcc, and encompassed 11 PFGE pulsotypes [13]. This clone was characterized by PCR positivity for both the *B. cepacia* epidemic strain marker and cable pilin, and showed close genetic relatedness to the epidemic strain *B. cenocepacia* CZ1 (ST32). *B. cenocepacia* ST858 and ST859 were isolated from sputum of a single chronically colonized patient. *B. stabilis* ST857 was also identified in only one patient. An interesting feature of strains BCC267 (ST858) and BCC269 (ST859) was the fact that results of evolutionary distances between concatenated nucleotide sequences of analyzed alleles were not in concordance with MLST scheme [13]. This analysis grouped ST858 and ST859 within *B. multivorans*, while by MLST they were designated as *B. cenocepacia* IIIA but as novel STs [12].

Considering the high transmissibility and peculiarity of *B. cenocepacia* ST856, the aim of this study was to evaluate its virulence potential. We also investigated the virulence potential of other novel STs identified in Serbian CF patients.

## Methods

### Bacterial strains

Bacterial strains used in this study originated from patients (*n* = 182) followed at the National Cystic Fibrosis Centre which is located at the Mother and Child Health Care Institute of Serbia “Dr. Vukan Cupic”. All of the strains were characterized previously by our research group [13].

Their genetic relatedness was determined by pulsed-field gel electrophoresis (PFGE) analysis of *Spe*I macrorestriction profiles. Fourteen different genotypes were identified and they belonged to four different MLST types: *B. cenocepacia* ST856 (11 genotypes), *B. cenocepacia* ST858 (one genotype), *B. cenocepacia* ST859 (one genotype) and *B. stabilis* ST857 (one genotype) [13]. A dendrogram was derived from the Ward linkage of correlation coefficients between PFGE patterns of different genotypes using SPSS cluster analysis software (IBM Corp. Released 2012. IBM SPSS Statistics for Windows, Version 21.0. Armonk, NY: IBM Corp.).

### Biofilm production

Biofilm production was assessed as described previously [14], with modifications. Briefly, one colony of the overnight culture of bacterial strains was adjusted to 0.5 McFarland turbidity standard. Wells of microtiter plates were filled with 180 μL of Luria Bertani (LB) medium. 20 μL aliquots of previously prepared bacterial suspension were added to each well. Blank wells contained LB medium only. The plates were incubated aerobically for 24 h at 37 °C. All isolates were tested in triplicate. *Pseudomonas aeruginosa* PAO1 and *Escherichia coli* DH5α were used as positive and negative controls, respectively. After the incubation, wells were emptied and washed three times with 300 μl of sterile physiological saline, and then dried at 65 °C. The plates were stained with 0.1% crystal violet (CV) (HiMedia, India). Thereafter, plates were emptied and dried at 65 °C. The dye bound to the adherent cells was resolubilized with 200 μl of ethanol-acetone mixture (80:20, *v*/v). The optical density was measured at 595 nm using Infinite M200 pro (Tecan, Switzerland). Based on the optical densities of bacterial biofilms, all strains were classified as follows:

OD ≤ ODc – no biofilm producer; ODc < OD ≤ 2xODc – weak biofilm producer; 2xODc < OD ≤ 4xODc – moderate biofilm producer; 4xODc < OD – strong biofilm producer; where ODc is an average value of blank OD with three standard deviations added [14].

### Biofilm production under stress conditions

Biofilm formation was investigated under different stress conditions including dynamic stress (100 rpm, at 37 °C), cold stress (growth at 12 °C), heat shock (growth at 42 °C), increased CO_2_ levels (5% CO_2_, at 37 °C in HERAcell 150, Thermo Scientific, USA). Additionally, two different pH values of LB medium (pH 6.8 and pH 7.45) were used for all tested conditions. Assessment of biofilm production was done as mentioned above. The statistical significance of differences in biofilm production for each stressor was determined in relation to biofilm production of given strain without stress (37 °C) was tested by Student’s *t*-test.

### Collagen- and fibronectin-binding assays

The wells of Maxisorb plates (Nunc, Roskilde, Denmark) were coated with type I collagen (from rat tail, BD Bioscience, New Jersey, United States) (100 μg/ml) or human fibronectin (Serva, Heidelberg, Germany) (100 μg/ml) for 16 h at 4 °C. The collagen-binding ability of the selected strains was tested according to Miljkovic and coauthors [15] while the ability of tested strains to bind to fibronectin was assayed as previously described by Ahmed and coauthors [16]. Average of six absorbance values per each strain for collagen- and fibronectin-binding was compared with those of the non-coated wells. *Escherichia coli* DH5α was used as a negative control, while laboratory strain *Lactococcus lactis* BGKP1 was used as a positive control.

### Proteolytic, gelatinase and elastase activity assay

Proteolytic activity was determined by plating 10 μl aliquots of the stationary-phase culture of each strain on medium containing skimmed milk (3%) and agar (1.5%). The plates were incubated at 37 °C for 48 h. Protease producers form transparent halo around the colonies.

Gelatinase activity of representative strains was assessed by previously described method [17]. Transparent halo around colonies was considered as positives for gelatinase production.

The elastase assay was done as described by Jacobson and coauthors [18].

### Mucoidy scoring system

Semiquantitative method for scoring mucoidy among selected strains was performed as previously described [19]. Briefly, mucoidy was assessed on Yeast Mannitol (YEM) agar containing 4 g/L mannitol and 0.5 g/L yeast extract and 1.5% agar. Bacteria were subcultured from a frozen stock by using a cotton swab to inoculate one third of a 25-ml YEM plate, streaked to yield individual colonies, and grown at 37 °C for 48 h. Mucoidy was defined as follows: nonmucoid, semi-mucoid and mucoid.

### Motility assays

Swimming motility was individually assessed for each isolate on plates containing tryptone (10 g/l), NaCl (5 g/l) and 0.3% (wt/vol) agar as previously described [20]. After 24 h the diameters of the colonies were measured. Isolates showing growth of less than or equal to 10 mm in diameter at 24 h were classified as nonmotile, while those with >10 mm were classified as motile.

Medium used for the twitching motility assay consisted of LB broth (10 g/L tryptone; 5 g/L yeast extract; 10 g/L NaCl) with 1% (*w*/*v*) agar. Twitching plates were briefly dried and strains were stab inoculated with a sharp toothpick to the bottom of the Petri dish from an overnight-grown LB agar (1.5%, *w*/*v*) plate. After incubation at 37 °C for 24 h, the zone of motility at the agar/Petri dish interface was measured.

### DNA manipulations

PCR screenings of 6 genetic determinants (*cepI*, *cepR*, *fliG*, *llpE*, *wbiI*, *bcscV*) associated with virulence were performed in accordance with published protocols [21] for representative strains belonging to each genotype of ST856 (eleven) and for ST857, ST858 and ST859. Representative PCR amplicons were selected and sequenced in order to confirm the specificity of the reaction. Sequencing was performed by the Macrogen DNA Sequencing Service (Amsterdam, Netherlands).

## Results

We have analyzed laboratory collection of 182 *B. cepacia* complex isolates from CF patients in our previous study. Based on PFGE analysis 14 different genotypes were identified. These genotypes belonged to four different MLST types: *B. cenocepacia* ST856 (11 genotypes), *B. cenocepacia* ST858 (one genotype), *B. cenocepacia* ST859 (one genotype) and *B. stabilis* ST857 (one genotype) [13].

**Fig. 1:**
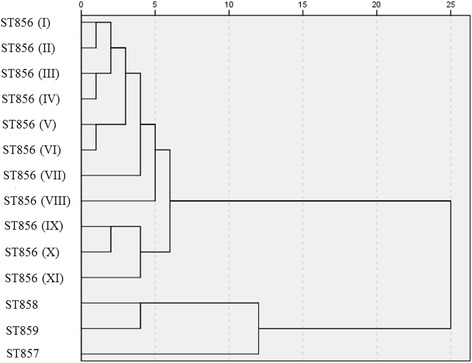
Dendrogram derived from *Spe*I PFGE patterns showing the relatedness of *Burkholderia cepacia* complex species isolated from Serbian CF patients. The dendrogram was constructed using SPSS software. Roman numerals indicate different genotypes of *B. cenocepacia* ST856 epidemic clone

Statistical analysis of PFGE results revealed that genetic differences among genotypes of the epidemic-related *B. cenocepacia* ST856 varied up to approximately 6% (Fig. 1). This variability pointed to the need of determination of virulence for all defined genotypes of ST856 epidemic clone (11 genotypes).

**Fig. 2:**
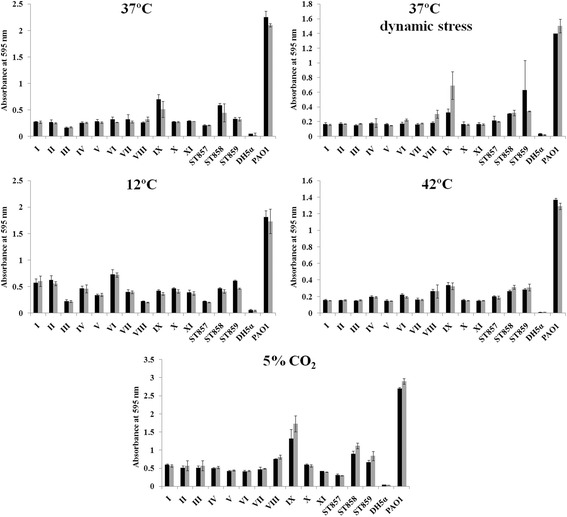
Biofilm formation of representative strains of different genotypes of *Burkholderia cepacia* complex isolated from Serbian CF patients under conditions that mimic milieu of CF lung and stress conditions. *Dark grey* histograms represent results for pH 6.8, *light grey* pH 7.45. Roman numerals indicate different genotypes of *B. cenocepacia* ST856 epidemic clone. Applied stress conditions are indicated in each graph. DH5α – negative control, PAO1 – positive control

Biofilm production was analyzed in all 182 isolates from the collection, and the majority of them were weak biofilm producers (74%) while only a small proportion produced strong biofilms (3%) (Table 1). Strong and moderate biofilm producers belonged to various ST856 genotypes, except moderate biofilm-producing strain BCC269 that belonged to ST859. Additionally, we compared biofilm formation capacity between isolates originating from chronically and transiently colonized patients. No statistically significant difference was observed between these two groups (*p* = 0.787 and *p* > 0.5, respectively) (Table 1). Since these results reflected biofilm production in standard laboratory conditions, we also analyzed biofilm production under stress conditions mimicking those faced by Bcc bacteria in the CF lung (changes in pH, dynamic stress, elevated CO_2_ levels) as well as impact of cold and heat shock for the selected representatives of each genotype (14 strains in total). We found that in all representatives of 11 *B. cenocepacia* ST856 genotypes cold stress (12 °C) and elevated CO_2_ levels (5% CO_2_) enhanced formation of biofilm with statistical significance (0.0001 < *p* < 0.005) (Fig. 2). Also, the heat stress (42 °C) and the dynamic stress reduced ability of biofilm formation in all representatives of ST856 genotypes with statistical significance (0.0001 < *p* < 0.005) (Fig. 2). Changes in pH values had no influence for all tested isolates (Fig. 2)*.* Biofilm formation of *B. cenocepacia* ST858 was reinforced under elevated CO_2_ levels (at both pH 6.8 and 7.45; *p* = 0.0041 and *p* = 0.0033, respectively) (Fig. 2). However, cold stress (pH 6.8, *p* = 0.0079), dynamic stress (pH 6.8, *p* = 0.0002) and heat shock (pH 6.8, *p* = 0.0002) reduced ability of ST858 to form biofilm. Other experimental conditions had no statistically significant impact on biofilm formation capacity of this strain (Fig. 2). *B. cenocepacia* ST859 more successfully formed biofilm under cold stress (at both pH 6.8 and 7.45; *p* < 0.0001 and *p* = 0.0023, respectively) and under elevated CO_2_ levels (at both pH 6.8 and 7.4; *p* = 0.0008 and *p* = 0.0029, respectively). Additionally, heat shock (at pH 6.8; *p* = 0.0293) reduced the ability of biofilm formation (Fig. 2). Other experimental conditions had no statistically significant influence on biofilm formation (Fig. 2). Biofilm formation in *B. stabillis* ST857 was enhanced by increased concentration of CO_2_ (at both pH 6.8 and 7.45; *p* = 0.0131 and *p* = 0.0002, respectively) although it was still poor, while other stressors had no statistically significant influence (Fig. 2).

**Table 1:** Results of biofilm formation compared to chronic or transient colonisation of the patients (number of isolates *n* = 182)

	Non-producers	Biofilm producers		
		Low	Moderate	Strong
Chronic colonisation (*n* = 161)	19	118	18	6
Transient colonisation (*n* = 21)	2	17	2	0
Total	21 (11.54%)	135 (74.18%)	20 (10.99%)	6 (3.29%)

Percentages are calculated relative to the total number of analysed strains

**Fig. 3:**
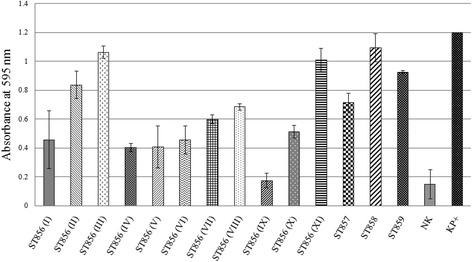
Collagen binding ability of *B. cenocepacia* ST856, *B. cenocepacia* ST858, *B. cenocepacia* ST859, and *B. stabilis* ST857. Roman numerals indicate different genotypes of *B. cenocepacia* ST856 epidemic clone. NK – *E. coli* DH5α, KP+ − *L. lactis* BGKP1

**Fig. 4:**
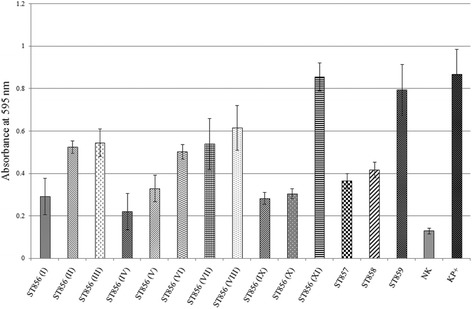
Fibronectin binding ability of *B. cenocepacia* ST856, *B. cenocepacia* ST858, *B. cenocepacia* ST859, and *B. stabilis* ST857. Roman numerals indicate different genotypes of *B. cenocepacia* ST856 epidemic clone. NK – *E. coli* DH5α, KP+ − *L. lactis* BGKP1

**Fig. 5:**
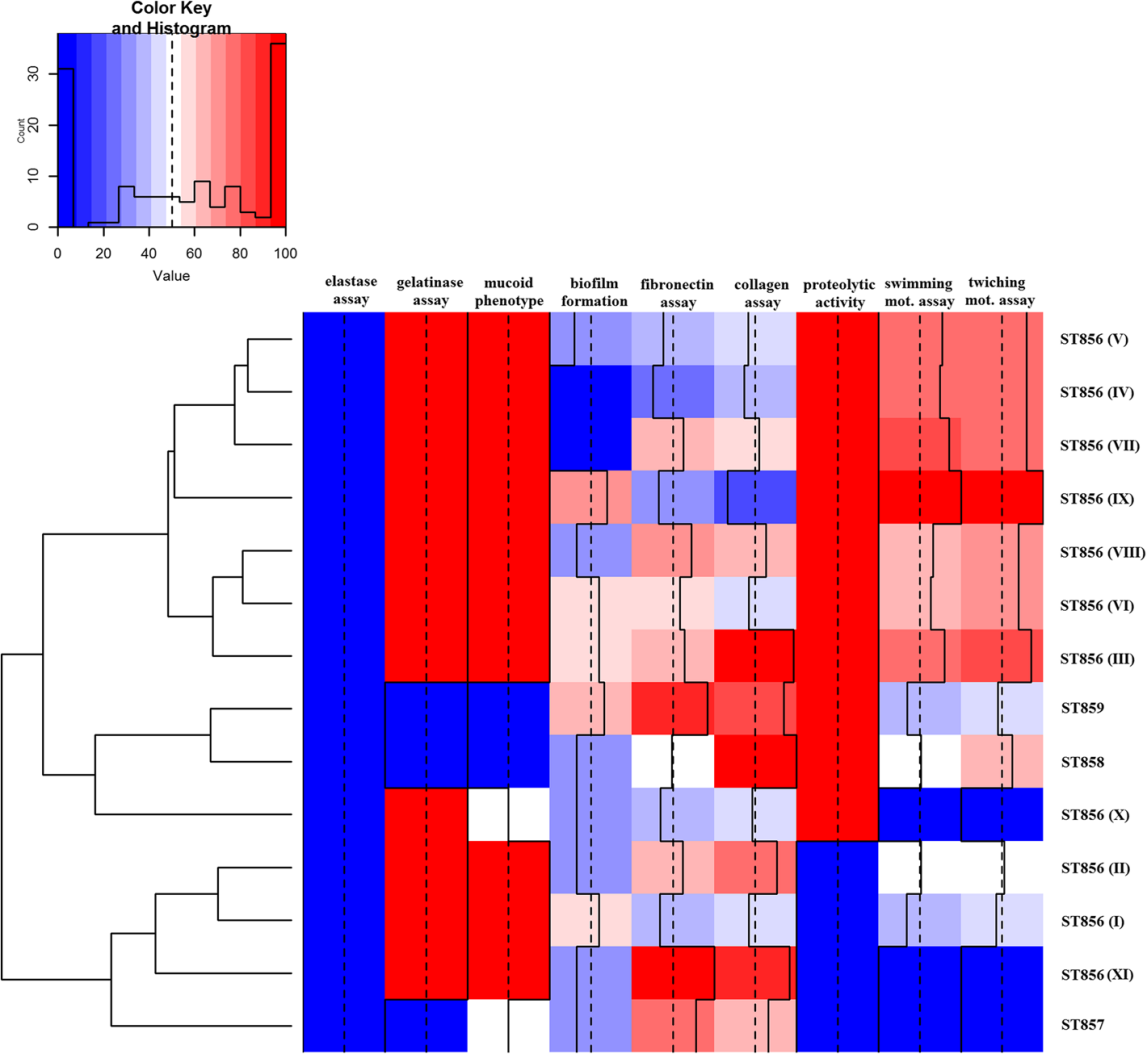
Heat-map describing presence of different virulence phenotypes of *B. cenocepacia* ST856, *B. cenocepacia* ST858, *B. cenocepacia* ST859, and *B. stabilis* ST857. Roman numerals indicate different genotypes of *B. cenocepacia* ST856 epidemic clone

Representatives of all genotypes from this study were tested for the ability to bind to collagen and fibronectin. It was noticed that strains adhered to immobilized collagen (Fig. 3) and fibronectin (Fig. 4) to different extents. Interestingly, differences in adherence to immobilized collagen and fibronectin were apparent between strains of different genotypes of *B. cenocepacia* ST856. In most strains, adherence to fibronectin and collagen correlated (Fig. 5).

Proteolytic activity testing, performed with skimmed milk, revealed that eight out of 11 of epidemic ST856 genotypes exhibited proteolytic activity (genotypes I, II, and XI did not form a transparent halo). *B. stabilis* ST857 was proteolytically inactive while *B. cenocepacia* ST858 and ST859 formed transparent halo, demonstrating proteolysis of milk proteins. None of the strains were able to hydrolyze elastine. Gelatinase activity was detected in all genotypes of ST856, while representatives of ST857, ST858, and ST859 were inactive in this assay (Fig. 5).

Mucoidy was tested by semiquantitative method in representatives of 14 genotypes. All genotypes of the ST856 clone were mucoid except genotype X that was semi-mucoid. *B. stabilis* ST857 was also semi-mucoid, while *B. cenocepacia* ST858 and ST859 were nonmucoid (Fig. 5).

Given the well-described correlation between the loss of swimming motility and establishment of chronic CF lung infections, we assessed swimming motility of bacteria of each genotype in order to understand the significance of swimming motility during Bcc infections. Nine out of 11 ST856 different genotype representatives were motile (zone of migration ranging from 12 to 35 mm), while genotypes X and XI were non-motile. *B. stabilis* ST857 was non-motile, whereas *B. cenocepacia* ST858 (18 mm) and ST859 (12 mm) could be considered as motile. Similar results were obtained for ST856 genotype representatives in twitching motility assay where nine out of 11 were positive (zone ranging from 17 to 40 mm). Genotypes X and XI were non-motile in twitching motility assay. *B. stabilis* ST857 was non motile, while *B. cenocepacia* ST858 (25 mm) and ST859 (18 mm) were motile.

Since quorum sensing, flagellum, some efflux-pumps and type-III secretion system were previously correlated with virulence, PCR screening for six genetic determinants (*cepI*, *cepR*, *fliG*, *llpE*, *wbiI*, and *bcscV*) was performed. Results revealed that *B. cenocepacia* ST856 strains and *B. stabilis* ST857 were positive for all analyzed genes. *B. cenocepacia* ST858 and ST859 were negative only for the *llpE* gene that is a part of the CeoAB-OpcM efflux pump operon.

Heat map demonstrating distribution of virulence characteristics analyzed in this study was constructed in order to summarize virulence potential of different genotypes of *B. cenocepacia* ST856, as well as *B. cenocepacia* ST858, ST859 and *B. stabilis* ST857 (Fig. 5). To compute a dendrogram, we used clustering algorithm that groups related rows together by similarity. Results were approximated on the relative scale ranging from 0 (blue) as the lowest value, progressing to white, then to 100 (red) as the highest value. Analyzed strains were separated into three clusters that reflect virulence potential. The heat-map revealed that gelatinase production, proteolytic activity, mucoidy, motility and adhesion to fibronectin and collagen were the most prominent characteristics of ST856 epidemic clone genotypes III, IV, V, VI, VII, VIII and IX strains were clustered together. ST857 was clustered with genotypes I, II and XI of ST856, since were phenotypically negative for most of the analyzed properties, with exception of adhesion to fibronectin and collagen and variable mucoidy appearance. ST859, ST858 and genotype X of ST856 were clustered together and on the basis of the positivity in applied phenotypic tests were grouped between previously mentioned clusters.

## Discussion

Considering the fact that in Serbian CF patients Bcc colonization prevalence reaches over 20% and that a single prevailing *B. cenocepacia* ST856 epidemic clone was identified in 96% of the patients infected with Bcc [13], the determination of its overall virulence potential has imposed as a potentially useful aid in understanding its successful dissemination and high prevalence. Divergence of 11 ST856 genotypes, which differed up to 6% according to PFGE analysis, raised the question of whether they possess common/distinct virulence potential. Among other virulence factors, ability to form biofilms is particularly intriguing since it contributes significantly to Bcc resistance to antimicrobials and antiseptics, resulting in treatment failure and persistence of infection [22]. Assessment of biofilm production in standard laboratory conditions revealed the low-level potential of ST856 genotypes. However, this observation may not reflect the real biofilm-forming potential of these bacteria, as in vivo bacterial biofilms are formed in a complex interaction with surrounding tissues and immune system of the host [23]. Additionally, novel findings indicate that Bcc bacteria are predominantely in the form of single cells or small clusters within phagocytes and mucus in late-stage CF lung, thus not forming biofilms [24]. These data suggest that Bcc bacteria have adapted as single-cell organisms, but not biofilms, to environments in the CF lung [24]. Thus formation of biofilm seems not to be crucial for pathogenicity of Bcc in CF lungs, and the low-level biofilm-forming potential of ST856 genotypes doesn’t mean that they are not well adapted for infection of CF-lungs. Previous reports concluded that *B. cenocepacia* strains, especially those belonging to genomovar III-A which encompasses ST856, ST858 and ST859 [12], could generally be considered avid biofilm producers [25]. Thus, it was of interest to determine whether the conditions that mimic the milieu of CF airways or additional stress conditions influence the potential for biofilm formation of these strains. Cold stress and increased concentration of CO_2_ enhanced biofilm formation by all ST856 genotypes indicating the possible significance of such experiments.

It has been previously postulated that the ability of Bcc to adapt and survive in acidic environment might be necessary for successful colonization of the CF lung due to fact that the pH of exhaled breath condensate is lower in patients with stable CF than in healthy controls, and is further reduced in CF patients with an infective exacerbation [26–28]. We demonstrated that, in the applied range, the pH value of the medium did not affect biofilm formation in analyzed ST856 genotypes. However, statistically significant influence of pH value on biofilm formation was detected in *B. cenocepacia* ST858 and ST859. These observations indicate that the response to decreased pH value is probably strain dependent. *B. stabilis* ST857 was shown to be a weak-biofilm producer, and this characteristic was not affected under different stress conditions except for increased concentration of CO_2_. This result is in accordance with the previously published results regarding this species [29]. Although all of the ST856 genotypes contained the *cepI-cepR* quorum sensing system, they exerted differences in the level of biofilm formation. This is not entirely surprising as correlation between the presence of quorum sensing and biofilm formation has been questioned previously. The strains lacking this correlation were described, such as *B. cenocepacia* ST28 with inactivated *cepI* autoinducer which was still able to form biofilm [29]. Molecular detection of virulence determinants revealed that *B. cenocepacia* ST858 and ST859 lacked the *llpE* gene encoding salicylate-regulated antibiotic efflux operon. This operon has been identified in *B. cenocepacia*, and the *llpE* gene is without parallel in previously reported efflux operons. The *llpE* gene was shown to be the most prevalent in *B. cenocepacia*, with a high degree of sequence conservation [7, 8]. The lack of this *B. cenocepacia* marker in genomes of ST858 and ST859 is particularly interesting in view of the unusual/unique phylogenetic status of these strains, as the results of evolutionary distances between concatenated nucleotide sequences of seven alleles that grouped the two STs close to *B. multivorans* were not in concordance with MLST scheme which identified them as *B. cenocepacia* [13].

Another virulence trait which may contribute to the severity of Bcc infections is the ability to secrete extracellular proteases, as reported for a number of Bcc species [30]. Proteolytic activity of the strains analyzed in this study varied among different genotypes of ST856, and 3/11 were proteolytically inactive. However, these results should be interpreted cautiously, as Bcc species could be phenotypically negative for proteolysis when skim milk agar is used as a substrate, even when the *zmpA gene encoding the most common extracellular protease of these bacteria is present in the genome* [30]. Mucoidy testing revealed that all genotypes of ST856 were mucoid, except genotype X that was semi-mucoid. This feature may contribute to the pathogenicity of the ST856 epidemic clone since the intensity of mucoidy was previously shown to be correlated with the rate of decline in pulmonary function as well as with increased virulence [31–33]. Also, mucoid isolates from CF patients had reduced interaction with macrophages and neutrophils enabling evasion of the host response [34]. However, it is worth noting that some exceptions exist, since all isolates of the virulent ST28 described to date are non-mucoid [19]. Additionally, it is known that Bcc strains change from a mucoid to a non-mucoid phenotype during chronic colonisation and patients which were infected exclusively with non-mucoid Bcc had a more rapid decline in lung function than those infected with mucoid bacteria [34].

## Conclusions

Our data suggest that detecting variations in virulence potential of different Serbian epidemic clone *B. cenocepacia* ST856 genotypes might be useful for risk assessment. Although all identified ST856 genotypes carry *cblA* and BCESM markers [13], phenotypic differences were observed regarding other virulence factors. This is not completely unexpected since *B. cenocepacia* is a member of a highly adaptable genus of bacteria that can rapidly evolve under in vitro stress conditions or during infections [35]. This adaptability may be the key in explaining differences observed among *B. cenocepacia* ST856 genotypes. Similarly, variability of virulence among the same genetic line for virulence was previously documented for the cable pili [35]. Diversity of epidemic clone genotypes virulence, could be challenging for accurate diagnosis and treatment, as well as for infection control. Thus, different genotypes of epidemic strains should be taken into account during decision making on cohort segregation of CF patients with Bcc infection.

## Abbreviations

Bcc: *Burkholderia cepacia* complex
CF: Cystic fibrosiss
BCESM: Burkholderia cepacia epidemic strain marker
MLST: Multilocus sequence typing
PFGE: Pulsed-field gel electrophoresis
LB: Luria Bertani
YEM: Yeast Mannitol
